# The effects of varying ingredients combination and boiling time on total phenolic content, antioxidant activity, and antimicrobial properties of lemongrass-ginger tea

**DOI:** 10.1016/j.heliyon.2024.e40172

**Published:** 2024-11-06

**Authors:** Aaron Dzigbor, David Neglo, Clement O. Tettey, Frank Nsaful, Elizabeth Owiredua Addo, Jennifer Ofosu-Pomaa

**Affiliations:** a Department of Food Science & Technology, Ho Technical University, Ho, Ghana; b Department of Biomedical Sciences, School of Basic & Biomedical Sciences, University of Health and Allied Sciences, Ho, Ghana; c Department of Food Process Engineering, School of Engineering Sciences, University of Ghana, Legon, Ghana

**Keywords:** Lemongrass-ginger tea, Antioxidant activity, Antimicrobial activity, Combinatory effect

## Abstract

This study was aimed at exploring the effect of varying lemongrass-ginger combinations, and boiling time on total phenolic contents (TPC), antioxidant activity, and antimicrobial efficacy of lemongrass-ginger tea. Lemongrass-ginger tea was produced by varying the percentage of lemongrass (25 %, 50 %, and 75 %) and boiling times (5, 10, and 15 min). The antioxidant activity of the lemongrass-ginger tea samples was investigated using the DPPH and ABTS assays whereas the TPC was determined using the Folin-Ciocalteau method. The antimicrobial activities were investigated by measuring the minimum inhibitory concentration (MIC), minimum bactericidal concentration (MBC), and minimum fungicidal concentration (MFC) of the tea against selected microorganisms, and its combinatory effects with antimicrobial drugs. The lemongrass-ginger combination and the boiling time significantly affected antioxidant potential, TPC, and antimicrobial activities. TPC measured ranged between 966.7 ± 90.20 to 1761.3 ± 81.70 μgGAE/g whereas DPPH antioxidant activities varied from 43.97 ± 14.99 % to 75.20 ± 8.55 %. The highest values of TPC and DPPH were 1761.3 ± 81.70 μgGAE/g and 75.20 ± 8.55 % and were recorded by 75 % lemongrass-ginger combination boiled for 15 min. Furthermore, differences in lemongrass-ginger combination and boiling times resulted in varying antimicrobial activities against the test microorganisms. The lowest MBC was recorded for 50 % lemongrass boiled for 10 min against *C. albicans*, 75 % lemongrass boiled for 15 min against *K. pneumoniae* and *S. typhi*, and 25 % lemongrass against *E. coli.* Additionally, varying ingredient proportions and boiling times affected the combinatory effect of the tea with antimicrobial drugs. However, the exact effect depends on the proportion of ingredients used and the boiling times.

## Highlights

• Proportion of ingredients and boiling time affected the total phenolic content.
• Antioxidant activity varied with the proportion of ingredients and boiling time.
• Antimicrobial activity depends on ingredient combination and boiling time.
• Interaction effects of combining herbal tea with antimicrobial drugs varied.

## 1 Introduction

Herbal tea is a beverage produced by either boiling or infusion of the leaves, flowers, seeds, fruits, stems, and roots of plant species other than *Camellia sinensis* leaves [1,2]. Herbal teas are consumed because of their therapeutic and health-promoting properties due to the active phytochemicals they contain [[1], [2], [3], [4], [5]]. Due to these benefits, the economic value of herbal tea consumption is on the rise. In 2022, the market value of herbal tea was estimated to be USD 3588.13 million and is expected to reach USD 4616.42 million by 2030 [6]. To meet the increasing demand for herbal tea, and changing consumer preferences it is necessary to find alternative plant sources or their combinations for herbal tea and evaluate their benefits. There are many studies involving lemongrass and ginger as one of the ingredients in literature [7,8] due to the several important health benefits of ginger, and lemongrass.

Ginger is abundant in active constituents, such as phenolic and terpene compounds. The major phenolic compounds in ginger include gingerols, shogaols, and paradols [9] which are responsible for the medicinal and biological activities of ginger [[10], [11], [12], [13], [14]], [15]]. In addition, ginger has been reported to contain flavonoids such as α-curcumene, α-zingiberene, β-sesquiphellandrene and β-bisabolene which may be responsible for medicinal and biological functions [[16], [17], [18]]. Lemongrass (*Cymbopogon citratus*) is a medicinal plant used for traditional herbal medicine as it contains bioactive compounds including alkaloids, flavonoids, saponins, quinones, tannins, carbohydrates, reducing sugar, and steroids [1,[19], [20], [21], [22], [23]]. Due to the presence of these bioactive compounds, lemongrass has been useful as an antimicrobial, antioxidant agent, a pain reliever, and for managing cough and cold [1,[24], [25], [26], [27]]. Lemongrass herbal tea was formulated using lemongrass, ginger powder, mint leaves powder, and cardamom powder [28]. Herbal tea was produced using ginger (*Zingiber officinale*) and *Pavetta crassipes* blends [29]. The therapeutic and medicinal benefits of these teas depend on the ingredients used in the formulation.

In Ghana, one of the popular herbal combines both lemongrass and ginger. This herbal tea is normally prepared at the household level by boiling crushed ginger and lemongrass in water. The proportion of lemongrass and ginger used, as well as the duration of boiling, varies widely from one person/household to another or even with the same person each time it is prepared. These variations could consequently, affect the biological and medical functionalities of the resulting tea. To help standardize the preparation process of this herbal tea, it is imperative to understand how the varying ingredient combinations and boiling times affect the functionalities of the tea. Therefore, this study aimed to investigate the effects of changing lemongrass-ginger combinations, and boiling time on the biological and medicinal properties of lemongrass-ginger tea. This was done by using different lemongrass-ginger combinations, and boiling times, and the effects of these changes on the resulting tea were analyzed by measuring the total phenolic content (TPC), antioxidant activity, and antimicrobial properties.

## 2 Materials and methods

### 2.1 Chemicals

The chemicals used in this study were DPPH (2,2 –diphenyl-1-picrylhydrazyl), methanol, ABTS (2, 2 -Azino-bis (3-ethylbenzthiazoline-6-sulphonic), potassium persulfate, sodium carbonate, Folin-Ciocalteu reagent, and gallic acid. All reagents were of analytical grade.

### 2.2 Source of sample materials

Fresh lemongrass leaves and ginger were used for this study. The lemongrass leaves and ginger were purchased from the Ho central market, and transported to the Department of Food Science and Technology for sample preparation and analysis.

### 2.3 Preparation of lemongrass-ginger tea

**Fig. 1:**
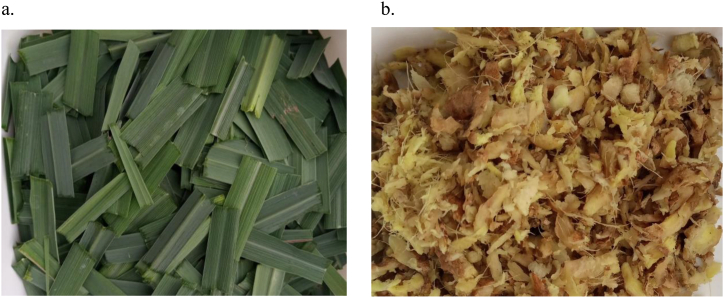
(a) Lemongrass leaves; (b) ginger used in the study.

The lemongrass leaves used were washed and cut into pieces (Fig. 1 (a)). The ginger was also washed and grated as shown in (Fig. 1 (b)). Different quantities of lemongrass leaves were accurately weighed and added to varying amounts of ginger such that the total mass of the lemongrass and ginger was 100 g. For the extraction, 500 ml of distilled water was measured into a glass beaker and boiled to a temperature of 100 °C using a heating mantle. A 100 g total mass of lemongrass-ginger combination was then added to the pre-boiled water and allowed to boil at 100 °C for further varying times. After this, the content of the beaker was transferred onto a cheesecloth and then squeezed to separate the liquid (tea) from the lemongrass-ginger residue. The resulting liquid was further filtered using Whatman no. 4 filter paper. The final filtrate was then poured into a ceramic bowl and evaporated at 40 °C in an oven to obtain a lemongrass-ginger tea extract powder. 1 g mass of the powdered tea extract was then dissolved in 2 mL of dimethyl sulfoxide (DMSO) to form a lemongrass-ginger tea extract solution which was used for further analysis. Tea extract from 100 g lemongrass only and 100 g ginger only were prepared in the same way to serve as the controls.

### 2.4 Experimental design

A 3^2^ experimental design was used in the study with the quantity of lemongrass leaves (hereafter referred to as lemongrass) and ginger varied from 25 g, 50 g, and 75 g such that the total mass of the lemongrass-ginger mixture was 100 g. The amount of lemongrass in the mixture was expressed as a percentage of the total mixture. For instance, 25 % lemongrass represents 25 g lemongrass and 75 g ginger. The boiling time for each sample mixture was varied. Thus, the independent variables used in this study and their levels were.

1. Percentage of lemongrass in the lemongrass-ginger at 25 %, 50 %, and 75 % levels
2. Boiling time at levels 5, 10, and 15 min.

In addition to this, lemongrass leaves only and ginger only were added as controls and their boiling times also varied for 5, 10, and 15 min (Table 1). The response variables measured in this study were total phenolic content (TPC), 2,2-diphenyl-1-picrylhydral (DPPH) activity, 2, 2-Azino-bis 3-ethylbenzthiazoline-6-sulphonic (ABTS) and antimicrobial activities. The experiments were conducted in triplicate.

**Table 1:** Experimental design of lemongrass-ginger tea.

Sample number	% of lemongrass to total mass of ginger and lemongrass	Boiling time (minutes)
1	25	5
2	25	10
3	25	15
4	50	5
5	50	10
6	50	15
7	75	5
8	75	10
9	75	15
Control	lemongrass only	5
Control	lemongrass only	10
Control	lemongrass only	15
Control	ginger only	5
Control	ginger only	10
Control	ginger only	15

### 2.5 Antioxidant activity determination

#### 2.5.1 DPPH radical scavenging activity

The DPPH radical scavenging activity of the lemongrass-ginger tea was measured in triplicate according to Dravie et al. [30]. A 40 μL of each herbal tea extract was added to 160 μL of 0.1 mM DPPH radical solution. The mixture was incubated at room temperature (25 °C) for 30 min. The absorbance was measured at 517 nm using Drawell DNM-9602 microplate reader.

The radical scavenging capacity was compared with that of ascorbic acid. The radical scavenging ability was determined as a percentage of the control sample using Equation (1):

1 $$\%\;inhibition=\frac{\left(\text{Ac}-\text{At}\right)}{\text{Ac}}\times 100$$

Where: Ac is the absorbance of control (in which the same volume of methanol was used in place of the sample); At is the absorbance of the samples and DPPH radical solution.

#### 2.5.2 ABTS radical scavenging activity

ABTS (2, 2 -Azino-bis (3-ethylbenzthiazoline-6-sulphonic) radical antioxidant activity of the lemongrass-ginger tea extracts was determined according to Dravie et al. [30]. 40 μL of each lemongrass-ginger tea extract was pipetted into microtiter plates in triplicates and 160 μL of ABTS radical solution was added to each sample. The mixtures were incubated in the dark for 30 min at room temperature (25 °C). Absorbance was read at 734 nm using Drawell DNM-9602 microplate reader. 200 μL of ABTS radical solution was pipetted in triplicates into microplates to be used as control. The radical scavenging capacity was compared with that of ascorbic acid. By subtracting the absorbance of lemongrass-ginger tea extracts from that of the absorbance of the free radical solution, the radical scavenging activity was determined as a percentage of the control sample using Equation (2):

2 $$\%\;inhibition=\frac{\left(\text{Ac}-\text{At}\right)}{\text{Ac}}\times 100$$

where: Ac is the absorbance of control; At is the absorbance of the samples and ABTS radical solution.

#### 2.5.3 Determination of total phenolic content (TPC)

Lemongrass-ginger tea extracts (100 μL) were added to 500 μL of Folin Ciocalteu's reagent and 1 mL sodium carbonate (20 % w/v). The mixture was incubated in the dark for 1 h at room temperature (25 °C). The absorbance was measured at 765 nm using Drawell DNM-9602 microplate reader. The values were interpolated using a standard gallic acid curve (0–0.3 mg/mL dissolved in water). From the gallic acid (GA) calibration curve (y=2.0927x+0.228), total phenolic content was quantified and expressed as micrograms of gallic acid equivalent per gram of sample (μgGAE/g). All experiments were performed in triplicates.

### 2.6 Antimicrobial activity

#### 2.6.1 Test organisms

The microorganisms used for the determination of antimicrobial activity were *Escherichia coli (*ATCC 25922), *Salmonella typhi (*ATCC 14028), *Klebsiella pneumoniae (NCTC 13440)*, and *Candida albicans (*ATCC 90028*).* These organisms were obtained from the Microbiology Laboratory, Department of Basic Sciences, School of Basic and Biomedical Sciences, University of Health and Allied Science (UHAS), Ghana. These organisms were chosen based on their involvement in the majority of infections and illnesses, and are also listed among World Health Organization's critical-priority pathogens because of their resistance to antibiotics [31,32].

#### 2.6.2 Determination of antimicrobial activity

The minimum inhibitory concentrations (MIC) of the lemongrass-ginger tea extract were evaluated to determine their antimicrobial properties. To determine the MIC of the test extracts, the micro broth dilution method was employed using the 96 well microtiter plates according to the protocol previously reported in the literature [33,34] with slight modifications. A 100 mg/mL stock solution was prepared by dissolving dried extracts of the lemongrass-ginger tea in 20 % DMSO. About 100 μL of double-strength Mueller Hinton broth (Oxoid Limited, United Kingdom) was pipetted into each 96-well plate and mixed with 100 μL of the lemongrass-ginger tea extract to create well concentrations ranging from 50.0 to 0.049 mg/mL using the stock solution, resulting in ten different concentrations. For each column in the microtiter plate, wells 11 and 12 were utilized as the positive control (broth + organism only) and negative control (broth with no organism), respectively, for each microbial strain. Similarly, separate plates were prepared for antibiotics, tetracycline, amoxicillin, fluconazole, and nystatin, and their concentrations were varied from 128.0 to 0.125 μg/mL, against all test bacteria and fungi. Subsequently, 100 μL of each of the 0.5 McFarland standardized test organisms was added after which the plates were incubated at 37 °C for 24 h for bacteria strains and 48 h for fungal strains. The MIC values were then evaluated by visual examination by adding tetrazolium chloride (TTC), 0.1 % (w/v) dye after 10 min and the MICs were determined as the lowest concentration which did not change colour from colourless/light yellow to red/pink.

#### 2.6.3 Minimum bactericidal (MBC) and minimum fungicidal concentration (MFC) determination

Minimum bactericidal/fungicidal concentrations (MBC/MFC) were determined to investigate whether the extracts of the lemongrass-ginger tea would be able to kill microbial cells. To do this, samples from each well of the susceptibility testing assays were transferred onto plates containing sterile nutrient agar and incubated at 37 °C for 24 h for bacteria strains and 48 h for fungal strains. The plates were then examined for the presence or absence of growth on the nutrient agar [35].

#### 2.6.4 Determination of synergistic effects of test samples and antibiotics

The effect of the lemongrass-ginger extract when combined with antimicrobial orthodox drugs was evaluated against the test microbes using the checkerboard method, with slight modifications based on previous studies by Khodavandi et al. [36] and Ankudze et al. [37]. Solutions with varying proportions of lemongrass-ginger extract and drugs (final volume of 200 μL) were prepared from twice the MIC solutions of each test sample and individual antibiotics (1 mg/mL). The antimicrobial activity of these solutions was determined, as described for MIC determination. The combinatory effect of the lemongrass-ginger extract when combined with antimicrobial orthodox drugs was then evaluated as a measure of the Fractional Inhibitory Concentration (FIC) index which was calculated using Equation (3) [37].

3 $$FIC\;Index=\left(\frac{MICA+S}{MICA}\right)+\left(\frac{MICS+A}{MICS}\right)$$

where MICA+S represents the lowest amount of antibiotics required in combination with lemongrass-ginger tea samples to inhibit bacterial growth, MICS+A represents the lowest concentration of lemongrass-ginger tea samples in combination with antibiotics. The minimum inhibitory concentrations of lemongrass-ginger tea samples and antibiotics are represented with MICS and MICA, respectively. The interaction between the antimicrobial orthodox drugs and the lemongrass-ginger tea samples is classified as:

i. synergistic if the FIC Index ≤0.5;
ii. partially synergistic if FIC index >0.5 and < 1;
iii. additive if the FIC Index = 1;
iv. No difference if the FIC Index is > 1 and ≤ 4; and
v. antagonistic if the FIC Index is > 4.0 [37].

### 2.7 Statistical analysis

Statistical analysis was done using one-way analysis of variance (ANOVA). A significant difference between the means was determined by Fisher's Least Significant Difference (LSD) procedure (p ≤ 0.05). The values reported are the means of triplicate determination ± standard deviation.

## 3 Results and discussion

### 3.1 Effect of preparation procedure on the total phenolic content of lemongrass-ginger tea

Total phenolic content (TPC) is one of the important parameters to be considered when evaluating the presence of polyphenols in extracts. The TPC measured ranged between 966.7 ± 90.20 μgGAE/g to 1761.3 ± 81.70 μgGAE/g, with the lowest value recorded for tea sample prepared by boiling mixture containing 50 % lemongrass (50 g of lemongrass and 50 g ginger) for 5 min, while the highest value was recorded for tea sample prepared by boiling 75 g lemongrass and 25 g of ginger for 15 min (Table 2). The TPC varied significantly with relative amounts of lemongrass and ginger as well as the boiling time (p ≤ 0.05) (Table 3). It was also observed that for the same relative amounts of lemongrass and ginger, the longer the boiling time higher the amount of TPC released in the tea samples (Table 2). The longer boiling time may have increased the solubility of analytes in the cellular matrix of both ginger and lemongrass in the hot water, leading to the release of the phenolic compounds [38]. Thus, controlling the relative amounts of lemongrass and ginger, as well as the boiling time significantly affected the TPC of the sample teas (Table 3). Thus, different TPC values observed when both boiling time and amount of ingredients changed could be due to the release of different types of bioactive in varying concentrations from both lemongrass and ginger. Lemongrass is known to contain bioactive compounds such as alkaloids, flavonoids, saponins, quinones, tannins, carbohydrates, reducing sugar and steroids [1,[19], [20], [21], [22], [23]] while ginger is reported to contain phenolic compounds such as gingerols, shogaols, and paradols [9], as well as flavaniods such as α-curcumene, α-zingiberene, β-sesquiphellandrene and β-bisabolene [18].

**Table 2:** Total phenolic content of lemongrass-ginger tea.

Sample Number	% lemongrass	Boiling time (minutes)	Total Phenolic Content (μgGAE/g fresh weight)
1	25	5	1274.8 ± 48.60^de^
2	25	10	1424.4 ± 120.80^cd^
3	25	15	1669.3 ± 160.30^ab^
4	50	5	966.7 ± 90.20^f^
5	50	10	1150.7 ± 26.30^e^
6	50	15	1541.3 ± 104.20^bc^
7	75	5	1215.6 ± 63.20^e^
8	75	10	1303.6 ± 32.30^de^
9	75	15	1761.3 ± 81.70^a^
Control	Lemongrass only	5	1318.0 ± 54.30
Control	Lemongrass only	10	1768.5 ± 88.30
Control	Lemongrass only	15	2201.0 ± 314.00
Control	Ginger only	5	1295.6 ± 32.70
Control	Ginger only	10	1913.0 ± 205.00
Control	Ginger only	15	2038.0 ± 559.00

Note: Values in the same column with different superscripts are significantly different at P ≤ 0.5 using Fisher's Least Significant Difference (LSD) procedure.

**Table 3:** Summary of ANOVA of the effects of % lemongrass and boiling time on TPC, DPPH, and ABTS.

Effect	Total Phenolic content		DPPH		ABTS	
	F-value	p-value	F-value	p-value	F-value	p-value
Intercept	6139.498	0.0000	1777.945	0.0000	2011.706	0.0000
% Lemongrass	18.202	0.0000	6.452	0.0077	47.871	0.0000
Time	74.323	0.0000	13.231	0.0003	8.263	0.0028
Lemongrass × Time	1.432	0.2638	0.735	0.5801	0.755	0.5676

These compounds could be released during the tea preparation. However, the type of the bioactive compounds, as well as the concentration could vary from one tea sample to another. at different times and in varying concentration. The values of TPC recorded in this study (Table 2) are less than TPC values of between 10.10 and 12.44 mgGAE/g recorded for a herbal tea involving lemongrass, ginger, and roselle [1]. These differences in value may be due to the tea preparation procedure and the presence of roselle, a third ingredient in their formulation.

### 3.2 Antioxidant activity of lemongrass-ginger tea

The antioxidant activity of the tea extracts was evaluated using two antioxidant assays commonly employed: DPPH and ABTS**.** Both the DPPH and ABTS antioxidant measurements varied significantly with relative amounts of lemongrass and ginger as well as the boiling time (p ≤ 0.05) (Table 3). Values of DPPH vary from 43.97 ± 14.99 % for tea prepared by boiling 25 % lemongrass for 5 min to 75.20 ± 8.55 % for tea prepared by boiling 75 % lemongrass for 15 min (Table 4). It was observed from Table 4 that, for the same amounts of lemongrass the longer the boiling time the higher the DPPH values recorded. This suggests that a longer boiling time may have caused bioactive compounds to leach out (Table 2) with more DPPH antioxidant capacity compared with a short boiling time. Similarly, it can be observed that for the same boiling time, as the amounts of ingredients changed the values of DPPH recorded also changed. This suggests that changes in the amounts of ingredients may have altered the type and concentration of bioactive compounds released during the herbal tea preparation. These bioactive compounds are responsible for the antioxidant activity [24,39]. Consequently, the DPPH values changed. Thus, controlling both the lemongrass mixture and boiling time can be used to control DPPH. The DPPH radical scavenging effects obtained in this study are comparable to those obtained by Suseno [1] who analyzed the antioxidant activity of a herbal tea produced from lemongrass, roselle, and ginger. It DPPH scavenging effect was found to be between 75 and 83 % [1]. The higher values of DPPH recorded in this study may be due to the roselle in their herbal tea. Aboagye et al. [4] conducted an experiment to determine the DPPH scavenging activity of fresh lemongrass leaves boiled in water for 15 min. They found that the inhibition was between 45 and 61 % [4], which is similar to those obtained for lemongrass in this study.

**Table 4:** Antioxidant and total polyphenol content of lemongrass-ginger tea.

Sample Number	% Lemongrass	Boiling time (minutes)	DPPH (%)	ABTS (%)
1	25	5	43.97 ± 14.99^e^	69.78 ± 6.68^b^
2	25	10	56.58 ± 1.73^cd^	56.92 ± 4.81^bc^
3	25	15	60.95 ± 4.99^bcd^	45.30 ± 9.60^c^
4	50	5	52.43 ± 8.16^a^	66.96 ± 3.71^b^
5	50	10	55.97 ± 2.22^cde^	62.93 ± 7.39^b^
6	50	15	65.51 ± 5.71^abc^	55.80 ± 7.10^bc^
7	75	5	52.77 ± 4.66^de^	96.12 ± 1.12^a^
8	75	10	70.01 ± 5.33^ab^	94.53 ± 2.31^a^
9	75	15	75.20 ± 8.55^a^	85.20 ± 4.75^a^
Control	Lemongrass only	5	56.32 ± 5.26	99.40 ± 0.81
Control	Lemongrass only	10	59.78 ± 1.76	97.41 ± 0.74
Control	Lemongrass only	15	63.08 ± 3.20	83.28 ± 17.17
Control	Ginger only	5	43.05 ± 4.16	42.62 ± 4.02
Control	Ginger only	10	53.59 ± 8.30	55.21 ± 4.19
Control	Ginger only	15	62.33 ± 1.66	54.73 ± 5.71

Note: Values in the same column with different superscripts are significantly different at P ≤ 0.5 using Fisher's Least Significant Difference (LSD) procedure.

Values of ABTS vary from 45.30 ± 9.60 % for tea prepared by boiling 25 % lemongrass for 15 min to 96.12 ± 1.12 % for tea prepared by boiling 75 % lemongrass for 5 min (Table 4). From the results, it can be seen that the changes in the relative amount of ingredients used and boiling time individually significantly influenced the ABTS activity of the tea extracts (Table 3). It could be observed from Table 4 that prolonged boiling time (for tea prepared with the same amount of lemongrass leaves) reduced the ABTS antioxidant activity of the tea extracts. This may be due to the thermal degradation of the polyphenols extracted in the tea samples [39]. Similarly, for the same boiling time, ratios of lemongrass and ginger used changed the ABTS scavenging activity of the tea extract. This may be because varying the amount of ingredients varied the type and concentration of the bioactive compounds released which consequently affected the antioxidant activity (Table 2, Table 4). The ABTS scavenging effects for tea prepared with lemongrass only (Table 4) are comparable to those obtained by Aboagye et al. [4] who determined ABTS activity of fresh lemongrass boiled in water for 15 min to be between 83 and 108 % [4].

### 3.3 Minimum inhibitory concentrations of lemongrass-ginger tea

To explore the possible antimicrobial properties of combined lemongrass and ginger extract, minimum inhibitory concentrations (MIC) studies against the growth of microorganisms were investigated**.** The results from this study, as presented in Table 5, Table 6, Table 7, Table 8 showed that extracts from tea prepared using different quantities of lemongrass and ginger demonstrated varying degrees of antimicrobial properties against *Escherichia coli* (ATCC 25922), *Klebsiella pneumoniae* (NCTC 13440), *Salmonella typhi (*ATCC 14028), and *Candida albicans* (ATCC 90028), respectively.

**Table 5:** Minimum inhibitory concentration of lemongrass-ginger tea against *Escherichia coli*.

Sample Number	%Lemongrass	Boiling time (Minutes)	MIC (μg/mL)	MBC (μg/mL)	MBC/MIC	Interpretation
1	25	5	6.25	12.50	2	Bactericidal
2	25	10	0.05	1.56	40	Bacteriostatic
3	25	15	6.25	12.50	2	Bactericidal
4	50	5	12.50	25.00	2	Bactericidal
5	50	10	6.25	25.00	4	Bactericidal
6	50	15	3.13	6.25	2	Bactericidal
7	75	5	12.50	12.50	1	Bactericidal
8	75	10	25.00	25.00	1	Bactericidal
9	75	15	1.56	3.13	2	Bactericidal
10	Lemongrass	5	25.00	25.00	1	Bactericidal
11	Lemongrass	10	3.13	6.25	2	Bactericidal
12	Lemongrass	15	12.50	25.00	2	Bactericidal
13	Ginger	5	6.25	25.00	4	Bactericidal
14	Ginger	10	0.05	1.56	40	Bacteriostatic
15	Ginger	15	12.50	50.00	4	Bactericidal

**Table 6:** Minimum inhibitory concentration of lemongrass-ginger tea against *Klebsiella pneumoniae*.

Sample Number	%Lemongrass	Boiling time (Minutes)	MIC (μg/mL)	MBC (μg/mL)	MBC/MIC	Interpretation
7	25	5	6.25	6.25	1	Bactericidal
8	25	10	50.00	50.00	1	Bactericidal
9	25	15	6.25	25.00	4	Bactericidal
1	50	5	12.50	12.50	1	Bactericidal
2	50	10	12.50	12.50	1	Bactericidal
3	50	15	3.13	6.25	2	Bactericidal
4	75	5	6.25	12.50	2	Bactericidal
5	75	10	3.13	3.13	1	Bactericidal
6	75	15	1.56	3.13	2	Bactericidal
10	Lemongrass	5	25.00	25.00	1	Bactericidal
11	Lemongrass	10	6.25	6.25	1	Bactericidal
12	Lemongrass	15	6.25	6.25	1	Bactericidal
13	Ginger	5	3.13	6.25	2	Bactericidal
14	Ginger	10	0.10	1.56	20	Bacteriostatic
15	Ginger	15	6.25	12.50	2	Bactericidal

**Table 7:** Minimum inhibitory concentration of lemongrass-ginger tea against *Salmonella typhi* (ATCC 14028).

Sample Number	%Lemongrass	Boiling time (Minutes)	MIC (μg/mL)	MBC (μg)/mL)	MBC/MIC	Interpretation
1	25	5	25.00	25.00	1	Bactericidal
2	25	10	0.16	6.25	40	Bacteriostatic
3	25	15	3.13	6.25	2	Bactericidal
4	50	5	25.00	25.00	1	Bactericidal
5	50	10	1.56	6.25	4	Bactericidal
6	50	15	1.56	6.25	4	Bactericidal
7	75	5	25.00	25.00	1	Bactericidal
8	75	10	12.50	25.00	2	Bactericidal
9	75	15	0.78	3.13	4	Bactericidal
10	Lemongrass	5	25.00	50.00	2	Bactericidal
11	Lemongrass	10	6.25	25.00	4	Bactericidal
12	Lemongrass	15	3.13	25.00	8	Bacteriostatic
13	Ginger	5	12.50	25.00	2	Bactericidal
14	Ginger	10	12.50	12.50	1	Bactericidal
15	Ginger	15	6.25	12.50	2	Bactericidal

**Table 8:** Minimum inhibitory concentration of lemongrass-ginger tea against *Candida albicans* (ATCC 90028).

Sample Number	%Lemongrass	Boiling time (Minutes)	MIC (μg/mL)	MFC (μg/mL)	MFC/MIC	Interpretation
1	25	5	3.13	3.13	1	Fungicidal
2	25	10	3.13	12.50	4	Fungicidal
3	25	15	3.13	12.50	4	Fungicidal
4	50	5	1.56	6.25	4	Fungicidal
5	50	10	25.00	50.00	2	Fungicidal
6	50	15	1.56	3.13	2	Fungicidal
7	75	5	25.00	50.00	2	Fungicidal
8	75	10	25.00	25.00	1	Fungicidal
9	75	15	12.50	25.00	2	Fungicidal
10	Lemongrass	5	3.13	3.13	1	Fungicidal
11	Lemongrass	10	3.13	3.13	1	Fungicidal
12	Lemongrass	15	12.50	50.00	4	Fungicidal
13	Ginger	5	3.13	3.13	1	Fungicidal
14	Ginger	10	0.10	3.13	40	Fungistatic
15	Ginger	15	6.25	12.50	2	Fungicidal

For instance, extracts from tea prepared by boiling 25 % lemongrass for 10 min demonstrated strongest antimicrobial activity against *Escherichia coli* (ATCC 25922) with MIC & MBC values of 0.04 μg/mL and 1.56 μg/mL while extract from tea produced by boiling 75 % lemongrass for 10 min showed the least ability to inhibit *Escherichia coli* (ATCC 25922) with MIC & MBC values of 25 μg/mL and 25 μg/mL, respectively (Table 5). Among the controls, extract from ginger only with MIC & MBC values of 0.04 μg/mL and 1.56 μg/mL showed the strongest antimicrobial activity against *Escherichia coli* (ATCC 25922) (Table 5). The variation in the MIC values could be due to differences in the type and concentration of bioactive compounds released from the ingredients during the preparation of the tea. Lemongrass contains several phenolic compounds such as gallic acid, isoquercetin, quercetin, rutin, catechin, and tannic acid [40] while ginger contains gingerols, shogaols, and paradols [9]. These compounds are reported to have antimicrobial activities [[10], [11], [12], [13], [14]], [15]]. These polyphenols alter the permeability of the cell, inhibit RNA synthesis, inhibit DNA gyrase as well as inhibit the enzymes required for the bacterial cell to produce energy [41]. Aboagye et al. [4] demonstrated that tea preparation time affects the release of bioactive. Thus, the differences in MIC values recorded could be due to the differences in the type and concentration of bioactive compounds released during tea preparation. Therefore, varying quantities of lemongrass and ginger to prepare tea at varying boiling times affect the antimicrobial properties of the resulting tea. Similar studies involving the determination of MIC value for ginger and lemongrass extract have been reported in the literature. For instance, an aqueous extract of ginger has been shown to possess antimicrobial properties with a MIC value of 0.1 mg/ml [42] while a methanolic extract of lemongrass has also been reported to possess antimicrobial activity with a MIC value of 1.5 mg/ml against *Escherichia coli* [43]. The lower MIC values obtained in this study may be due to the presence of bioactive compounds with strong activities against *Escherichia coli*. The antimicrobial properties of lemongrass-ginger tea against *Klebsiella pneumoniae* (NCTC 13440) are presented in Table 6. Extracts from tea produced by boiling 75 % lemongrass for 15 min were most effective against *Klebsiella pneumoniae* (NCTC 13440) with MIC and MBC values of 1.56 μg/mL and 3.13 μg/mL, respectively, while extract from tea sample prepared by boiling 25 % lemongrass for 10 min recorded the least activity against *Klebsiella pneumoniae* (NCTC 13440) with MIC and MBC values of 50 μg/mL*.* Among the controls, ginger only (control) with MIC & MBC values of 0.10 μg/mL and 1.56 μg/mL showed the strongest antimicrobial activity against *Klebsiella pneumoniae* (NCTC 13440). Previous studies reported successful inhibition of *Klebsiella pneumoniae* has been documented in literature. It has been demonstrated that aqueous ginger extract has antibacterial properties with a MIC value of 0.25 mg/mL [42], while lemongrass methanolic extract has antimicrobial activity against *Klebsiella pneumoniae* with a MIC value of 1.5 mg/mL [43]. The extract from this herbal tea is more effective against *Klebsiella pneumoniae* than the literature values, as seen by the greater literature values when compared to the MIC values in Table 6.

The susceptibility of *Salmonella typhi* (ATCC 14028) when tested against the various tea samples is presented in Table 7. Extract from tea sample produced by boiling 25 % lemongrass for 10 min demonstrated the strongest activity against *Salmonella typhi* (ATCC 14028) with an MIC value of 0.16 μg/mL whilst *Salmonella typhi* (ATCC 14028) was least susceptible tea extracts were 25 % lemongrass for boiled for 5 min, 50 % lemongrass boiled for 5 min, and 75 % lemongrass boiled for 5 min with same MIC value of 25 μg/mL (Table 7). Among the controls, an MIC value of 3.13 μg/mL was obtained for the extract from lemongrass only boiled for 15 min whilst an MIC value of 25 μg/mL was recorded for lemongrass only boiled for 5 min (Table 7). Tea samples prepared by boiling ginger alone in this study showed the strongest activity against *Salmonella typhi* compared with an MIC value of 0.25 mg/mL obtained by Gull et al. [42] using aqueous ginger extract against *Salmonella typhi* (ATCC 14028). This may be due to the presence of bioactive compounds with strong activities against *Salmonella typhi* (ATCC 14028).

In terms of minimum bactericidal concentration (MBC), 75 % lemongrass boiled for 15 min demonstrated the strongest ability to destroy *Salmonella typhi* (ATCC 14028) microorganisms with an MBC value of 3.13 μg/mL whilst the sample of the tea with the least ability to destroy microorganism were 25 % lemongrass for boiled for 5 min, 50 % lemongrass for boiled for 5 min, 75 % lemongrass boiled for 5 min and 75 % lemongrass boiled for 10 min and with same MBC value of 25 μg/mL (Table 7). The inhibitory and fungicidal properties of the various tea samples as presented in Table 8 showed different tea samples with different minimum inhibitory and fungicidal values. For instance, extracts from tea samples prepared by boiling 50 % lemongrass for 5 min and 50 % lemongrass for 10 min demonstrated the strongest inhibitory activity with MIC value of 1.56 μg/mL, whilst extracts from tea samples produced by boiling 50 % lemongrass for 10 min, 75 % lemongrass for 5 min and 75 % lemongrass for 10 min showed the least inhibitory activity against *Candida albicans* (ATCC 90028) with MIC value of 25 μg/mL.

Among the controls, ginger only boiled for 10 min had the lowest MIC value of 0.10 μg/mL. The MIC values of the tea samples against *Candida albicans* (ATCC 90028) were lower than the MIC value of 50 mg/mL obtained by Ajijolakewu et al. [21] to inhibit *Candida albicans*. This suggests the tea samples have bioactive compounds with strong activity against *Candida albicans.* In addition, extracts from 25 % lemongrass boiled for 5 min and 50 % lemongrass boiled for 15 min with the same MFC value of 3.13 μg/mL showed the strongest fungicidal activity against *Candida albicans* (ATCC 90028), while 75 % lemongrass boiled for 5 min had the least fungicidal activity with MFC value of 50 μg/mL (Table 8). Further insights into the mode of action of the tea extracts were obtained by computing the MBC/MIC ratio (for bacteria strains) or MFC/MIC ratio (for fungal strains). The interpretation of these ratios revealed that apart from 75 % lemongrass boiled for 10 min which was bacteriostatic against *Escherichia coli* (ATCC 25922) (Table 5) and *Salmonella typhi* (ATCC 14028) (Table 7), all the other test samples showed varying degrees of bactericidal property (for bacteria strains) (Table 5, Table 6, Table 7) and fungicidal property (for fungal strain) (Table 8). Thus, sample 8 would help prevent the growth of bacteria by keeping the growth of bacteria in a stationary phase (bacteriostatic), while the other samples would destroy the bacteria (bactericidal) or fungi (fungicidal). The bactericidal effect may be attributed to the release of bioactive compounds during the hot decoction process (Table 2) which can penetrate the internal structures and interact with specific molecules within the microbial cells, leading to their death. These results indicated that different efficacy levels against a particular pathogen could be obtained if the lemongrass-ginger tea is prepared by following different preparation conditions. In a nutshell, the results presented in Table 5, Table 6, Table 7, Table 8 showed that the relative amount of ingredients and boiling time affected the tea sample's efficacy against the test organisms, with different test organisms exhibiting variable degrees of susceptibility. This may be attributed to extra polymeric compounds, flagella, extracellular structures, and surface hydrophobicity which are claimed to affect how different microorganisms react to various antimicrobial treatments [37].

### 3.4 Synergistic effect of lemongrass-ginger tea in combination with orthodox antimicrobial drugs

There is an upsurge in antimicrobial resistance of pathogens against orthodox antimicrobial agents [37]. Thus, there is a need for studies to enhance the antimicrobial efficacy of orthodox antimicrobial agents. In this study, lemongrass-ginger teas were combined with standard antimicrobial agents (amoxicillin, tetracycline, fluconazole, and nystatin). The results of this study are presented in Table 9, Table 10, Table 11.

**Table 9:** Synergistic effect of tetracycline and sample lemongrass-ginger tea on test bacteria.

Sample Number	%Lemongrass	Boiling time (Minutes)	*Escherichia coli (*ATCC 25922)		*Klebsiella pneumoniae (NCTC 13440)*		*Salmonella typhi (*ATCC 14028)	
			FIC Index		FIC Index		FIC Index	
			Sample + Tetracycline	Interpretation	Sample + Tetracycline	Interpretation	Sample + Tetracycline	Interpretation
7	25	5	1.50	ND	1.12	AD	1.13	AD
8	25	10	2.00	ND	1.13	AD	1.13	AD
9	25	15	2.00	A	5.00	A	5.00	A
1	50	5	0.37	S	0.14	S	0.56	PS
2	50	10	0.75	PS	2.12	ND	3.00	ND
3	50	15	0.19	S	0.14	S	4.50	A
4	75	5	0.37	S	1.12	AD	1.13	AD
5	75	10	0.38	S	1.25	AD	1.12	AD
6	75	15	0.25	S	0.27	S	1.13	AD
10	Lemongrass	5	2.00	ND	5.00	A	2.50	ND
11	Lemongrass	10	0.50	S	0.62	PS	0.75	PS
12	Lemongrass	15	0.12	S	0.19	S	0.16	S
13	Ginger	5	2.50	ND	8.50	A	4.50	A
14	Ginger	10	2.51	A	4.50	A	8.50	A
15	Ginger	15	1.50	ND	0.56	S	0.56	S

Note: A represents Antagonistic; S represents Synergistic; AD represents Additive; ND represents No Difference; PS represents Partially Synergistic.

**Table 10:** Synergistic effect of amoxicillin and sample lemongrass-ginger tea on test bacteria.

Sample Number	%Lemongrass	Boiling time (Minutes)	*Escherichia coli (*ATCC 25922)		*Klebsiella pneumoniae (NCTC 13440)*		*Salmonella typhi (*ATCC 14028)	
			FIC Index		FIC Index		FIC Index	
			Sample + Amoxicillin	Interpretation	Sample + Amoxicillin	Interpretation	Sample + Amoxicillin	Interpretation
7	25	5	0.23	S	0.63	PS	0.63	PS
8	25	10	1.00	AD	0.19	S	0.56	PS
9	25	15	0.25	S	0.44	S	0.37	S
1	50	5	0.37	S	0.63	PS	0.63	PS
2	50	10	1.00	AD	0.63	PS	0.63	PS
3	50	15	1.00	AD	0.63	PS	0.75	PS
4	75	5	1.00	AD	0.63	PS	0.63	PS
5	75	10	1.00	AD	0.63	PS	0.63	PS
6	75	15	1.00	AD	0.88	PS	0.88	PS
10	Lemongrass	5	0.25	S	0.22	S	0.22	S
11	Lemongrass	10	2.00	ND	0.31	S	0.75	PS
12	Lemongrass	15	1.50	ND	0.31	S	0.75	PS
13	Ginger	5	0.37	S	0.87	PS	0.88	PS
14	Ginger	10	0.76	PS	0.63	PS	0.63	PS
15	Ginger	15	0.50	S	0.18	S	0.63	PS

Note: A represents Antagonistic; S represents Synergistic; AD represents Additive; ND represents No Difference; PS represents Partially Synergistic.

**Table 11:** Synergistic effect of antifungal agents (nystatin and fluconazole) and sample lemongrass-ginger tea on *Candida albicans (*ATCC 90028*)*.

Sample Number	%Lemongrass	Boiling time (Minutes)	FIC Index			
			Sample + Nystatin	Interpretation	Sample + Fluconazole	Interpretation
7	25	5	8.69	A	2.50	ND
8	25	10	3.00	A	2.50	ND
9	25	15	12.00	A	1.25	AD
1	50	5	22.03	A	2.50	ND
2	50	10	7.00	A	1.00	AD
3	50	15	2.50	ND	0.52	PS
4	75	5	3.00	ND	2.50	ND
5	75	10	2.50	ND	2.50	ND
6	75	15	2.12	ND	2.50	ND
10	Lemongrass	5	10.00	A	1.25	AD
11	Lemongrass	10	1.50	ND	0.75	PS
12	Lemongrass	15	2.03	ND	2.50	ND
13	Ginger	5	4.50	A	2.50	ND
14	Ginger	10	2.50	ND	1.25	AD
15	Ginger	15	3.01	ND	0.63	PS

Note: A represents Antagonistic; S represents Synergistic; AD represents Additive; ND represents No Difference; PS represents Partially Synergistic.

Various degrees of efficacy were observed when the tea samples were combined with the standard antimicrobial agents. The type of efficacy when the tea samples were combined with a particular antimicrobial agent depends on the tea sample being combined with the antimicrobial agent, and the type of microorganism on which it is being tested. For instance, there was a synergy when tetracycline was combined with extract from 50 % lemongrass boiled for 5 min and tested against *Escherichia coli* (ATCC 25922) and *Klebsiella pneumoniae* (NCTC 13440) and partial synergy when tested against *Salmonella typhi* (ATCC 14028) (Table 9). Similarly, there was no difference in efficacy when extract from 75 % lemongrass boiled for 5 min was combined with tetracycline and tested against *Escherichia coli* (ATCC 25922), but partially synergistic effect was observed when the same combination was tested against *Klebsiella pneumoniae* (NCTC 13440) and *Salmonella typhi* (ATCC 14028) (Table 10). Noor [43] reported the additive effect of lemongrass methanolic extract combined with tetracycline against both *Escherichia coli* and *Klebsiella pneumoniae*. In addition, a synergistic effect was observed when extracts from 50 % lemongrass boiled for 5 min and 75 % lemongrass boiled for 5 min were combined with amoxicillin and tested against *Escherichia coli* (ATCC 25922), whilst partial synergy was recorded when the same combinations were tested against *Klebsiella pneumoniae* (NCTC 13440) and *Salmonella typhi* (ATCC 14028) (Table 10). A similar synergy was reported by Noor [43] against *Escherichia coli* when amoxicillin was combined with methanolic lemongrass extract. Furthermore, when extract from 50 % lemongrass boiled for 5 min was combined with both nystatin and fluconazole, an additive effect and no difference in efficacy level were, respectively, observed when tested against *Candida albicans* (ATCC 90028) (Table 11). A similar combinatory effect was observed when extract from 75 % lemongrass boiled for 5 min was combined with both nystatin and fluconazole against the same fungus. These results also indicated that when different tea samples combined with the same standard antimicrobial agents and tested against a particular microorganism would produce different efficacy levels. Therefore, tea preparation conditions (relative amount of ingredients and boiling time) are very critical to obtain the same efficacy level.

## 4 Conclusion

The objective of this research was to explore the effect of varying relative proportions of lemongrass and ginger as well as boiling time on TPC, antioxidant, and antimicrobial properties of lemongrass-ginger tea. It was found from the study the variation in quantity of both lemongrass and ginger as well as the boiling time significantly affected the TPC and antioxidant activity of lemongrass-ginger tea. The various lemongrass-ginger teas also produced varying degrees of antimicrobial efficacy when tested against some selected pathogens. Thus, consumption of lemongrass-ginger tea may produce different efficacy levels if it is not prepared the same way all the time. Further analysis revealed that various combinatory effects could be produced when lemongrass-ginger tea prepared at varying preparation conditions is combined with standard antimicrobial agents. Thus, the consumption of a standard antimicrobial agent together with lemongrass-ginger tea could produce synergy, additive, antagonism, or no effect on efficacy level when the preparation condition for lemongrass-ginger tea changes.

## CRediT authorship contribution statement

**Aaron Dzigbor:** Writing – original draft, Supervision, Formal analysis, Conceptualization. **David Neglo:** Investigation. **Clement O. Tettey:** Writing – review & editing, Investigation. **Frank Nsaful:** Writing – review & editing, Supervision. **Elizabeth Owiredua Addo:** Investigation. **Jennifer Ofosu-Pomaa:** Investigation.

## Limitations and recommendation

The focus of the current study was to determine the effects of varying lemongrass-ginger combinations and boiling times on the total phenolic content and biological activities of the resulting tea. This is necessary to see how each variable of lemongrass-ginger tea preparation influences the functionalities of the tea. Therefore, the optimization of the lemongrass-ginger tea was not undertaken in this study. Furthermore, it is possible that varying the lemongrass-ginger combinations would affect the sensory attributes of the resulting tea. However, this was also not evaluated in the current research. Hence, it is recommended that further research be undertaken to optimize the lemongrass-ginger tea preparation to obtain the right lemongrass-ginger combinations and boiling times that give the best tea in terms of functionalities and sensory attributes, leading to standardized lemongrass-ginger herbal tea for industrial production.

## Declaration of competing interest

The authors declare that they have no known competing financial interests or personal relationships that could have appeared to influence the work reported in this paper.
